# Predictive utility of the baseline Tp–e interval for early arrhythmic events in acute myocardial infarction: A cohort study

**DOI:** 10.1177/03000605261452586

**Published:** 2026-06-10

**Authors:** Sreeja Gandamsetty, Shashikala Taggarshe Surkunda, Anupa Appu Shetty, Divya D Pai, Souvik Chaudhuri, Barkur Ananthakrishna Shastry

**Affiliations:** 1 Department of General Medicine, Kasturba Medical College, 76793 Manipal Academy of Higher Education, Manipal, Karnataka, India; 2 Department of Critical Care Medicine, 76793 Kasturba Medical College, Manipal Academy of Higher Education, Manipal, Karnataka, India

**Keywords:** Acute myocardial infarction, arrhythmia, T wave peak–T wave end interval, Tp–e/QT ratio, ST-elevation myocardial infarction, non-ST-elevation myocardial infarction

## Abstract

**Objective:**

Acute myocardial infarction is a major cause of early cardiovascular morbidity and mortality, with arrhythmias contributing to adverse outcomes. This study evaluated the predictive utility of the baseline T wave peak–T wave end interval for arrhythmic events within 48 h in patients presenting with the first episode of acute myocardial infarction.

**Methods:**

In this analytical cohort study, 196 patients with acute myocardial infarction were enrolled. Clinical characteristics, electrocardiographic findings, echocardiographic findings, angiographic findings, arrhythmias, complications, and in-hospital mortality were recorded.

**Results:**

The mean age of the patients was 62.1 ± 10.3 years, and 69.4% were male. Arrhythmias occurred in 65.3% of patients, most commonly ventricular tachycardia (21.8%). Multivariable logistic regression identified higher Killip class (III–IV) and prolonged T wave peak–T wave end interval as the independent predictors of arrhythmias. A T wave peak–T wave end cutoff ≥98 ms showed a sensitivity of 77.3%, specificity of 52.9%, and diagnostic accuracy of 69%. The T wave peak–T wave end and T wave peak–T wave end/QT ratios were significantly higher among nonsurvivors, along with greater clinical severity.

**Conclusion:**

A prolonged baseline T wave peak–T wave end interval (≥98 ms) and higher Killip class independently predict early arrhythmias in acute myocardial infarction.

## Introduction

Ischemic heart disease, particularly acute myocardial infarction (MI), is a leading cause of morbidity and mortality worldwide, with the highest risk concentrated within the first 48 h after symptom onset.^1,2^ During this vulnerable period, ischemia-induced myocardial injury results in marked electrical instability, predisposing patients to arrhythmias ranging from benign ectopy to life-threatening arrhythmias that significantly contribute to early post-MI mortality.^3^

Earlier studies have focused on QT interval–based indices to assess arrhythmic risk and predict clinical outcomes in critically ill patients.^4–6^ However, QT prolongation alone fails to capture the spatial and temporal heterogeneity of ventricular repolarization underlying malignant arrhythmias.^7^ This limitation has driven interest in electrocardiographic (ECG) markers reflecting regional repolarization dispersion.

Arrhythmogenesis in the early post-MI phase is multifactorial, involving ischemia-related conduction slowing, autonomic imbalance, and heterogeneity in myocardial recovery.^8,9^ Transmural dispersion of repolarization (TDR), which reflects differences in the duration of action potential across the ventricular wall, plays a pivotal role in creating a substrate for re-entrant arrhythmias.^10^ Experimental and clinical studies have demonstrated that the T wave peak–T wave end (Tp–e) interval is closely correlated with TDR and serves as a practical surface ECG marker of repolarization heterogeneity.^11,12^

Importantly, a substantial proportion of Asian patients experience adverse outcomes following MI despite lacking traditional risk factors, underscoring the need for improved early risk stratification.^13^ The Tp–e interval and Tp–e/QT ratio are significant predictors of arrhythmic or mortality outcomes in several cardiovascular diseases, including long QT syndrome, cardiomyopathy, heart failure, and MI.^12,14^

This study aimed to evaluate the utility of the Tp–e interval in predicting early arrhythmia risk within 48 h in patients presenting with the first episode of acute MI, thereby addressing the existing knowledge gaps to enhance early risk stratification, guide timely interventions, and improve survival outcomes. The primary objective was to determine the predictive value of the baseline Tp–e interval for early arrhythmia during the initial 48 h of hospitalization, while the secondary objective was to identify early clinical and investigational variables in patients with acute MI that significantly differ between survivors and nonsurvivors.

## Materials and methods

### Study design and setting

This analytical cohort study was conducted at Kasturba Hospital, Manipal, a tertiary care teaching hospital in southern India. The study was conducted over a period of 14 months, from February 2024 to March 2025. Approval was obtained from the Kasturba Medical College and Kasturba Hospital Institutional Ethics Committee (IEC)-2 (Student Research) (IEC number: IEC2: 31/2024, approval date: 20 November 2023) and the Clinical Trials Registry of India (CTRI) (registration number: CTRI/2024/01/061723). This study was conducted in accordance with the Helsinki Declaration of 1975, as revised in 2024. The reporting of this study conforms to the Strengthening the Reporting of Observational Studies in Epidemiology (STROBE) guidelines.^15^

### Study population and sample size

Adults aged ≥18 years presenting within 24 h of symptom onset with the first episode of acute MI were included. Diagnosis was established according to the Fourth Universal Definition of Myocardial Infarction,^16^ defined by a rise or fall in high-sensitivity cardiac troponin-T above the 99th percentile with at least one ischemic feature. Written informed consent was obtained from all participants, with surrogate consent obtained in cases of hemodynamic instability.

Patients with prior MI, chronic heart failure (New York Heart Association NYHA ≥II), previous coronary artery bypass surgery, structural or valvular heart disease, cardiomyopathy, congenital heart disease, channelopathies, baseline conduction abnormalities, recent use of class I or III antiarrhythmic or QT-prolonging drugs, implanted cardiac devices, pregnancy, active infection or sepsis, autoimmune disease, malignancy, chronic obstructive pulmonary disease (COPD) (Global Initiative for Chronic Obstructive Lung Disease or GOLD ≥II), thyroid dysfunction, symptomatic anemia (Hb <10 g/dL), severe renal impairment (eGFR <30 mL/min/1.73 m^2^), or unwilling to consent were excluded.

The sample size was estimated using the standard formula for sensitivity with specified precision:

$$n=\frac{Z^{2}\times \text{Sensitivity×}\left(1-\text{Sensitivity}\right)}{d^{2}\times \text{Prevalence}}$$

where S  = anticipated sensitivity, P =prevalence of the condition (proportion of true positives), d  = desired precision, and $\;Z_{1-\alpha /2}\;$ = Z score for 95% confidence. After adjusting for a 10% attrition rate, the final sample size was 196.

### Data collection

Overall, 196 eligible patients were enrolled consecutively and followed up during hospitalization. All patient details were deidentified to ensure confidentiality. The data for each patient were collected using a pretested, structured proforma. The baseline clinical and demographic information, including age, sex, history of hypertension, diabetes mellitus, substance use status, and Killip class at presentation,^17^ was recorded. Laboratory investigations included peak troponin-T and NT-proBNP levels within 24 h of admission, serum electrolytes (potassium, calcium, and magnesium), lipid profile, and serum creatinine. An echocardiographic (ECHO) evaluation was performed within 24 h of presentation to assess left ventricular ejection fraction (LVEF) and regional wall motion abnormalities. Coronary angiography findings were documented during hospitalization. All patients were continuously monitored in the intensive care unit for the first 48 h for arrhythmia detection and followed up until hospital discharge to record the clinical outcomes.

Arrhythmias were defined as any sustained or nonsustained supraventricular or ventricular tachyarrhythmia, bradyarrhythmia, or conduction abnormality occurring after acute MI, as described by Frampton et al.^3^ The ECG parameters were obtained from the baseline electrocardiogram recorded at presentation. The QT interval was measured from the onset of the QRS complex to the end of the T wave and corrected for heart rate using Bazett’s formula (corrected QT (QTc) interval). The Tp–e interval was measured in lead V_5_ (or V_4_/V_6_ when V_5_ was unsuitable) as the interval from the peak of the T wave to its end, determined by the intersection of the terminal downslope tangent with the isoelectric baseline (tangential method). The Tp–e/QT ratio was calculated from the same cardiac cycle. Two trained observers independently performed all the ECG measurements using digital calipers to ensure accuracy. ECGs with indistinct T wave endpoints, excessive noise, or pacing spikes were excluded. In cases of acute ST-segment elevation or depression, the measurements were obtained only when both the T wave peak and end were clearly identifiable. Patients with a new-onset bundle branch block were included if the T wave was measurable. Interobserver variability assessed in a random subset of 20 ECGs showed a mean difference of <5 ms for Tp–e measurements.

The clinical complications were defined as follows: pulmonary edema as acute dyspnea with bilateral crepitations and radiographic evidence of pulmonary congestion; hemodynamic instability as sustained hypotension (systolic blood pressure (SBP) < 90 mmHg or mean arterial pressure (MAP) <65 mmHg) requiring vasopressor support; and acute kidney injury (AKI) according to Kidney Disease: Improving Global Outcomes (KDIGO) criteria, defined by a rise in the value of serum creatinine ≥0.3 mg/dL within 48 h or ≥1.5 times baseline within 7 days.

The primary outcome was the predictive value of the Tp–e interval for arrhythmias within 48 h of hospitalization following the first MI. The secondary outcomes included variables differentiating survivors from nonsurvivors.

### Data analysis

Microsoft Excel and SPSS (version 27) were used for data entry, cleaning, and analysis. Continuous variables were presented as means ± standard deviations (SDs), and categorical variables were summarized as frequencies and percentages. The Kolmogorov–Smirnov test was used to assess the normality of continuous variables. The Mann–Whitney U test was used to assess the change in the means across independent variables. The chi-square test or Fisher’s exact test (if the expected count was <5) was used to assess the associations between the variables. For predicting arrhythmia in patients with acute MI, univariate analysis was performed, and the variables that were found to be significant were selected for the multivariable logistic regression analysis. The adjusted odds ratio and 95% confidence interval for the independent predictors of arrhythmia were calculated. A receiver operating characteristic curve (ROC) analysis was performed to assess the area under the curve (AUC) of significant continuous variables after regression analysis. The cutoff value was selected based on the point that provided an optimal balance between sensitivity and specificity, along with the positive predictive value, negative predictive value, and diagnostic accuracy. Statistical significance was determined at the 5% level. AI-assisted tools (Grammarly, Rubriq) were used solely for language editing and grammar correction. No AI tools were used for data analysis, interpretation, or generation of scientific content.

## Results

### Demographic, comorbidity, and lifestyle characteristics of the study population

The study population had a mean age of 62.1 ± 10.3 years. Most patients belonged to the 51–60 (34.2%) and 61–70 (31.1%) year age groups, while only 1.0% were aged 31–40 years and 4.1% were aged 81–90 years. Males predominated (69.4%), with females comprising 30.6% of the cohort. Hypertension (60.7%), dyslipidemia (58.7%), and diabetes mellitus (44.4%) were the most prevalent comorbid conditions. Smoking was reported by 71.9% of patients, while alcohol consumption was noted in 34.7%. A family history of coronary artery disease was noted in 3.1% of participants.

### ECG, ECHO, and angiographic findings of the study population

An ECG analysis revealed a mean PR interval of 160.18 ± 32.79 ms, QRS duration of 104.79 ± 50.07 ms, QT interval of 398.07 ± 56.58 ms, and QTc of 466.98 ± 86.17 ms. Repolarization indices showed a mean Tp–e interval of 111.94 ± 31.06 ms and a Tp–e/QT ratio of 0.29 ± 0.10.

Echocardiography demonstrated no regional wall motion abnormality (RWMA) in 23 patients, while 147 had 1 RWMA, 15 had 2 RWMAs, and 3 had 3 RWMAs. LVEF was >40% in 150 patients and <40% in 46 patients. Coronary angiography revealed normal coronary arteries in 7 patients, single-vessel disease in 120 patients, double-vessel disease in 60 patients, and triple-vessel disease in 9 patients.

### Clinical characteristics and outcomes of the study population

Chest pain was the most common presenting symptom (82.1%, n = 161), followed by dyspnea (20.9%, n = 41) and palpitations (8.2%, n = 16). ST-elevation MI (STEMI) was observed in 79.6% (n = 156) of the patients, and non-ST-elevation MI (NSTEMI) was observed in 20.4% (n = 40) of the patients. Arrhythmias occurred in 65.3% (n = 128) of the patients. The most frequent arrhythmias were ventricular tachycardia (VT) (21.8%, n = 28), sinus bradycardia (20.3%, n = 26), sinus tachycardia (14.8%, n = 19), complete heart block (CHB) (12.5%, n = 16), atrial fibrillation (AF) (7.8%, n = 10), ventricular premature complexes (VPCs) (7.0%, n = 9), and first/second-degree AV block (5.5%, n = 7). Less common abnormalities included ventricular fibrillation (n = 4), new-onset left bundle branch block (LBBB) (n = 3), new-onset right bundle branch block (RBBB) (n = 3), accelerated idioventricular rhythm (AIVR) (n = 2), and junctional rhythm (n = 1).

Among patients with arrhythmias, 78% (n = 100) developed arrhythmias within the first 6 h, whereas 15 and 13 patients experienced arrhythmias at 6–12 h and 12–48 h, respectively. The complications included pulmonary edema (26%, n = 51), cardiogenic shock (29%, n = 57), AKI (32.1%, n = 63), and in-hospital mortality (8.1%, n = 16). VT was the most frequent arrhythmia among nonsurvivors (56.3%, 9/16) followed by sinus tachycardia (18.8%, 3/16), VF, CHB, VPC, and AF. The distributions of the arrhythmia subtypes by the time of onset, ejection fraction, and survival status are presented in Supplementary Tables 1, 2, and 3, respectively.

### Comparison of ECG indices across clinical subgroups

The descriptive statistics of the Tp–e interval and Tp–e/QT ratio are presented in Supplementary Table 4. These indices did not differ significantly based on the age group, sex, comorbidities, substance use, or STEMI versus NSTEMI presentation. However, patients with a higher Killip class, as shown in Supplementary Figure 1A and 1B, and those with reduced LVEF (<40%), as shown in Supplementary Table 4, demonstrated significantly prolonged Tp–e intervals and elevated Tp–e/QT ratios. Patients with arrhythmias had a significantly higher Tp–e interval (p < 0.001), Tp–e/QT ratio (p < 0.001), and QTc interval (p = 0.002) than those without arrhythmias.

### Demographic, clinical, and outcome differences according to arrhythmia status

Patients who developed arrhythmias were significantly older than those without arrhythmias (p = 0.016), and age showed a significant association with arrhythmia occurrence (χ^2^ = 10.69, p = 0.03) (Supplementary Table 5A and 5B). Smoking was significantly associated with arrhythmia development (p = 0.02), while hypertension, diabetes mellitus, dyslipidemia, and alcohol consumption were not. A higher Killip class showed a strong association with arrhythmia occurrence (p < 0.001). Cardiogenic shock, pulmonary edema, and AKI were significantly more frequent in patients with arrhythmias (p < 0.001). Mortality occurred exclusively in the arrhythmia group, as shown in Table 1.

**Table 1.** Descriptive statistics of the arrhythmia and nonarrhythmia groups.

Variable	Arrhythmia (128)	Nonarrythtmia (68)	p value
Age (years; mean and SD)	63.43 ± 10.14	59.72 ± 10.23	**0.016**
Sex (female)	41 (32%)	19 (27.9%)	0.554
Diabetes mellitus	54 (42.2%)	33 (48.5%)	0.395
Hypertension	77 (58.6%)	44 (64.7%)	0.404
Dyslipidemia	72 (56.3%)	43 (63.2%)	0.344
Smoking	99 (77.3%)	42 (61.8%)	**0.021**
Alcohol	42 (32.8%)	26 (38.2%)	0.448
STEMI	106 (82.8%)	50 (73.5%)	0.125
LVEF <40%	42 (32.8%)	4 (5.9%)	**<0.001**
Killip I & II	86 (69.1%)	68 (100%)	**<0.001**
Killip III & IV	42 (30.9%)	0	**<0.001**
QTc (milliseconds)	478.82 ± 98.36	444.69 ± 49.99	**0.002**
Tp–e interval (milliseconds)	121.00 ± 31.42	94.91 ± 22.07	**<0.001**
Tp–e to QT ratio	0.31 ± 0.10	0.25 ± 0.06	**<0.001**
Pulmonary edema	45 (35.2%)	6 (8.8%)	**<0.001**
Shock	51 (39.8%)	6 (8.8%)	**<0.001**
AKI	56 (43.8%)	7 (10.3%)	**<0.001**
Death	16 (12.5%)	0	**0.002**

AKI: acute kidney injury; LVEF: left ventricular ejection fraction; ms: milliseconds; QTc: corrected QT interval; SD: standard deviation; STEMI: ST-elevation myocardial infarction.

### Predictors of arrhythmia

In the univariate analysis, the variables significantly associated with arrhythmia were older age (p = 0.018), smoking (p = 0.022), reduced LVEF (<40%; p < 0.001), higher Killip class (III/IV; p < 0.001), QTc interval (p = 0.011), prolonged Tp–e interval (p < 0.001), elevated Tp–e/QT ratio (p < 0.001), and adverse outcomes such as pulmonary edema (p < 0.001), cardiogenic shock (p < 0.001), and AKI (p < 0.001). Multivariable logistic regression identified higher Killip class (III/IV; adjusted OR: 12.93, p = 0.015) and prolonged Tp–e interval (adjusted OR: 1.024, p = 0.003) as the independent predictors of arrhythmia. Although both the Tp–e interval and Tp–e/QT ratio were significant predictors in the univariate analysis, only the Tp–e interval was included in the multivariable model. As the Tp–e interval is an integral component of the Tp–e/QT ratio, the inclusion of both variables would introduce redundancy and strong interdependence. Similarly, Tp–e was not included alongside QTc in the multivariate regression model, as it represents the terminal component of ventricular repolarization and is derived from the QT interval, making it inherently correlated with QTc. Including these closely related variables could result in unstable coefficient estimates, inflated standard errors, and reduced interpretability of their independent effects. Therefore, the Tp–e interval alone was used to represent repolarization dispersion in the final model. Univariate and multivariable regression analyses are illustrated in Table 2.

**Table 2.** Univariate analysis and multivariable logistic regression for the prediction of arrhythmia.

Variables	Univariate analysis			Multivariable logistic regression		
	p value	OR	95% CI	p value	Adjusted OR	95% CI
Age	0.018	1.037	1.006–1.068	0.091	1.031	0.995–1.068
Smoking	0.022	2.113	1.114–4.011	0.135	1.824	0.829–4.015
LVEF <40%	<0.001	7.782	2.653–22.827	0.277	2.063	0.558–7.718
QTc(ms)	0.011	1.005	1.001–1.009			
Killip class III/1V	<0.001	31.575	4.234–235.449	**0.015**	12.928	1.632–102.432
Tp–e (ms)	<0.001	1.035	1.022–1.048	**0.003**	1.024	1.008–1.039
Tp–e/QT	<0.001	2.811	1.790–4.415			
Pulmonary edema	<0.001	5.625	2.252–14.048	0.175	2.243	0.698–7.212
Cardiogenic shock	<0.001	6.892	2.769–17.154	0.731	0.791	0.208–3.013
AKI	<0.001	6.841	2.897–16.152	0.219	2.022	0.657–6.218

AKI: acute kidney injury; CI: confidence interval; LVEF: left ventricular ejection fraction; ms: milliseconds; OR: odds ratio.

### Diagnostic performance of the Tp–e interval

**Figure 1.**
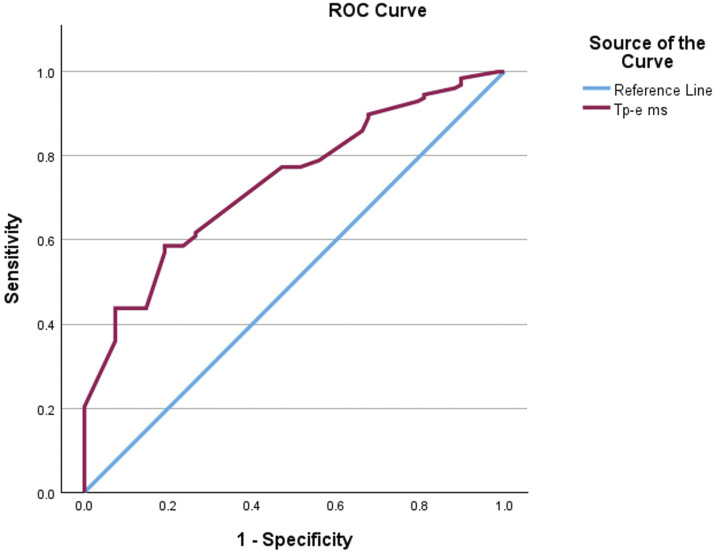
ROC analysis of the Tp–e interval for predicting arrhythmias. ROC: receiver operating characteristic, Tp–e ms: Tp–e interval in milliseconds.

ROC curve analysis demonstrated that a Tp–e interval cutoff of ≥98 ms predicted arrhythmia with an AUC of 0.736, as shown in Figure 1 and Supplementary Table 6. At this threshold, the overall diagnostic accuracy was 69%, with a positive likelihood ratio of 1.64, indicating fair discriminative ability, as shown in Table 3.

**Table 3.** Diagnostic performance of the Tp–e interval for predicting ventricular arrhythmias.

Variable	Arrhythmia absent	Arrhythmia present
Tp–e < 98 ms	36 (55.4%)	29 (44.6%)
Tp–e ≥ 98 ms	32 (24.4%)	99 (75.6%)

Chi-square test, p < 0.001. Sensitivity: 77.3%, specificity: 52.9%, diagnostic accuracy: 69%, positive predictive value: 75.6%, negative predictive value: 55.4%, positive likelihood ratio: 1.64, and negative likelihood ratio: 0.43

ms: milliseconds.

### Comparison between survivors and nonsurvivors

Among the 196 patients studied, 16 (8.2%) did not survive. Nonsurvivors had significantly higher Tp–e intervals and Tp–e/QT ratios, with a greater prevalence of reduced LVEF (<40%), higher Killip class (III/IV), arrhythmias, pulmonary edema, cardiogenic shock, and AKI compared with survivors, as presented in Table 4.

**Table 4.** Differences in the parameters between survivors and nonsurvivors.

Variables	Survivors (n = 180)	Nonsurvivors (n = 16)	p value
Age (years)	61.84 ± 10.42	65.50 ± 8.39	0.174^a^
Tp–e (ms)	110.29 ± 30.60	130.57 ± 31.15	**0.012** ^a^
Tp–e/QT	0.283 ± 0.096	0.347 ± 0.083	**0.005** ^a^
QTc (ms)	468.77 ± 86.94	446.91 ± 76.54	0.332^a^
LVEF <40%	31 (17.4%)	15 (93.8%)	**<0.001^b^**
Smoking	127 (70.6%)	14 (87.5%)	0.244^b^
Killip class 3/4	34 (18.9%)	8 (50%)	**0.004^c^**
AKI	52 (29.7%)	11 (68.8%)	**0.001^c^**
Cardiogenic shock	42 (24%)	15 (93.5%)	**<0.001^c^**
Pulmonary edema	41 (23.4%)	10 (62.5%)	**<0.001^c^**
Hypertension	113 (62.8%)	6 (37.5%)	0.062^c^
Diabetes mellitus	80 (44.4%)	7 (43.8%)	0.957^c^
Dyslipidemia	105 (58.3%)	10 (62.5%)	0.746^c^
Alcohol consumption	60 (33.3%)	8 (50%)	0.180^c^
STEMI	141 (78.3%)	15 (93.8%)	0.143^c^
Arrhythmia present	112 (62.2%)	16 (100%)	**0.002^c^**

AKI: acute kidney injury; LVEF: left ventricular ejection fraction; ms: milliseconds; QTc: corrected QT interval; SD: standard deviation; STEMI: ST-elevation myocardial infarction.

a Independent Student’s t test; ^b^Fisher’s exact test; ^c^Chi-square test

### Association of beta-blocker and reperfusion therapy with outcomes

Treatment data were available for 186 of the 196 patients. Treatment-related analyses are provided in Supplementary Tables 7–10. The absence of beta-blocker therapy was significantly associated with a higher incidence of arrhythmias (p = 0.003). Beta-blocker use was significantly associated with lower mortality (p < 0.001). Reperfusion therapy did not show a significant association with arrhythmias (p = 0.680) or mortality (p = 0.271).

## Discussion

The present study highlights the prognostic significance of ventricular repolarization indices in acute MI. Evaluation of these parameters in a real-world cohort underscores their practical applicability as bedside tools for identifying high-risk patients. Patients with advanced clinical severity and greater electrical dispersion, reflected by a higher Killip class and prolonged Tp–e interval, were more likely to experience arrhythmic events. This observation suggests that both hemodynamic compromise and electrical instability contribute to the risk of arrhythmia.

Older age significantly predicted post-MI arrhythmias in our study, consistent with the study by Evren Dal and Suna Eraybar,^18^ while sex and comorbidities showed no association, aligning with the results of Oikawa and Özbek et al.^19^ Smoking independently predicted arrhythmias, consistent with the study by Kurmi et al.^20^ Most arrhythmias occurred within 6–12 h, corroborating the findings of Marangmei et al.^21^ and Hamid Khederlou et al.;^22^ The STEMI–NSTEMI rates were similar, in contrast to the findings of Henkel et al.^23^ and Xu et al.^24^

Clinical severity at presentation strongly influenced the risk of arrhythmia. A higher Killip class (III–IV) was associated with a markedly increased risk of arrhythmia (OR: 31.575, 95% CI: 4.234–235.449, p < 0.0001). This finding is consistent with reports by Henkel et al.,^23^ Oikawa et al.,^19^ and Xu et al.,^24^ although Bendary et al.^25^ did not observe a similar association. Reduced left ventricular systolic function also emerged as a key predictor; patients with LVEF <40% had a significantly higher risk of arrhythmia (OR: 7.782, 95% CI: 2.653–22.827, p < 0.0001). These findings reinforce the established link between impaired myocardial function, increased electrical instability, and arrhythmogenesis in acute MI.

A prolonged Tp–e interval reflects an increased dispersion of ventricular repolarization, predisposing patients to arrhythmias, particularly in the setting of myocardial ischemia.^10,11^ A key observation of this study is the significant difference in the Tp–e interval and Tp–e/QT ratio between patients with and without arrhythmias. Similar observations have been reported by Özbek and Sökmen^26^ and Yu et al.^7^ Together, these results reinforce the role of repolarization heterogeneity as an important electrophysiological determinant of arrhythmogenesis in patients with acute myocardial ischemia.

In the multivariable logistic regression analysis, the Tp–e interval emerged as an independent predictor of arrhythmia (adjusted OR: 1.024, p = 0.003), along with a higher Killip class (adjusted OR: 12.93, p = 0.015). These findings are concordant with prior studies in patients with STEMI. Bendary et al.^25^ reported a similar magnitude of risk (OR: 1.023, p = 0.033), while Özbek and Sökmen^26^ demonstrated a stronger association (OR: 1.05, p < 0.001). Beyond acute coronary syndromes, Tp–e prolongation has been shown to predict arrhythmic events in Takotsubo syndrome (OR: 1.07, p = 0.011) by La Rosa et al.^27^ and in Brugada syndrome (OR: 9.61, p < 0.0001) by Maury et al.^28^ A meta-analysis by Tse et al.^29^ further identified Tp–e prolongation (mean cutoff: 103.3 ± 17.4 ms) as a significant predictor of arrhythmic and mortality outcomes (OR 1.14, p < 0.001). Collectively, these data reinforce the role of Tp–e as a robust marker of ventricular repolarization heterogeneity across diverse clinical settings.

In the present study, ROC analysis identified a Tp–e interval cutoff of ≥98 ms for predicting ventricular arrhythmias (AUC: 0.736), with a diagnostic accuracy of 69%. The positive likelihood ratio of 1.64 suggests a modest but clinically relevant increase in arrhythmic risk, supporting Tp–e as a useful noninvasive risk stratification marker. Similar discriminatory performance has been reported across different arrhythmogenic conditions, albeit with variable cutoff values. Shenthar et al.^30^ reported that Tp–e >100 ms was associated with ventricular fibrillation in patients with STEMI, demonstrating high sensitivity and negative predictive value but limited specificity. In Brugada syndrome, Maury et al.^28^ reported that a maximum Tp–e >100 ms in any precordial lead was associated with ventricular arrhythmic events, demonstrating high sensitivity (84%) and excellent negative predictive value (98%). Similarly, Zumhagen et al.^31^ identified a Tp–e cutoff ≥77 ms with moderate sensitivity (63.6%) and specificity (74.1%).

Other studies reported higher Tp–e thresholds with more modest performance. Azarov et al.^11^ demonstrated that the Tp–e interval predicted delayed ventricular fibrillation with an AUC of 0.800 and optimal cutoff of 123 ms (sensitivity 70%, specificity 73%). In Takotsubo syndrome, La Rosa et al.^27^ reported that a corrected global Tp–e at 48 h showed good discrimination (AUC 0.740), with a cutoff of 108 ms (sensitivity 71%, specificity 72%). Demidova et al.^32^ identified a global Tp–e cutoff of 131 ms for reperfusion-related ventricular fibrillation (AUC: 0.657; sensitivity: 58%, specificity: 73%). Tp–e shows fair-to-good discrimination across arrhythmogenic conditions but should be interpreted contextually. Tp–e, along with the Tp–e/QT ratio, may serve as a reliable, noninvasive marker of increased arrhythmic risk in acute myocardial ischemia.

In the present study, the nonsurvivors demonstrated a significantly prolonged Tp–e interval, higher Tp–e/QT ratio and Killip class, and reduced LVEF. Consistent with this, Erikssen et al.^33^ reported longer Tp–e intervals and lower LVEF among nonsurvivors, while Özbek and Sökmen^26^ showed significant Tp–e prolongation in patients with fatal ventricular arrhythmias. Collectively, prolonged Tp–e indices, reduced LVEF, higher Killip class, and arrhythmias cluster in nonsurvivors, reflecting shared pathophysiology of severe myocardial injury, electrical instability, and hemodynamic compromise driving increased mortality.

Although our study was not designed to evaluate the effects of treatment, we observed that the absence of beta-blocker therapy was associated with a higher incidence of arrhythmias, and beta-blocker use was associated with improved survival. These findings should be interpreted with caution due to the observational nature of the analysis, low number of mortality events, and potential confounding factors.

The main limitations include the single-center design, modest sample size, single-time-point ECG assessment, and short-term in-hospital follow-up. Treatment-related analyses were not part of the primary study design and were limited by incomplete data and potential confounding factors, restricting causal inference. However, our study had certain strengths. We enrolled a sizable cohort of 196 patients with acute MI and applied multivariable logistic regression to control for confounders, revealing that only advanced Killip class and prolonged Tp–e interval (≥98 ms) remained independent predictors of early arrhythmias. Importantly, the analysis was restricted to arrhythmic events within the first 48 h post-infarction, which is a period of maximal electrical vulnerability, enhancing the clinical relevance of the findings.

## Conclusion

In our study, prolongation of the Tp–e interval and advanced Killip class emerged as independent predictors of early arrhythmias in patients with acute MI. A Tp–e value >98 ms demonstrated fair discriminatory ability and may serve as a simple, noninvasive ECG marker for early arrhythmic risk stratification, particularly in resource-limited settings, facilitating timely identification of high-risk patients and potentially reducing morbidity and mortality.

## Supplemental Material

sj-pdf-1-imr-10.1177_03000605261452586 - Supplemental material for Predictive utility of the baseline Tp–e interval for early arrhythmic events in acute myocardial infarction: A cohort study Supplemental material, sj-pdf-1-imr-10.1177_03000605261452586 for Predictive utility of the baseline Tp–e interval for early arrhythmic events in acute myocardial infarction: A cohort study by Sreeja Gandamsetty, Shashikala Taggarshe Surkunda, Anupa Appu Shetty, Divya D Pai, Souvik Chaudhuri and Barkur Ananthakrishna Shastry in Journal of International Medical Research

## Data Availability

All the data analyzed, which support the findings of this study, are available from the corresponding author upon reasonable request.
