# Supplementary material for: Predictive utility of the baseline Tp–e interval for early arrhythmic events in acute myocardial infarction: A cohort study
Source: J Int Med Res. 2026 Jun 10;54(6):03000605261452586. doi: 10.1177/03000605261452586 (PMC13254428; doi:10.1177/03000605261452586)
Supplement: sj-pdf-1-imr-10.1177_03000605261452586 - Supplemental material for Predictive utility of the baseline Tp–e interval for early arrhythmic events in acute myocardial infarction: A cohort study [file sj-pdf-1-imr-10.1177_03000605261452586.pdf]

Supplementary Figure and tables

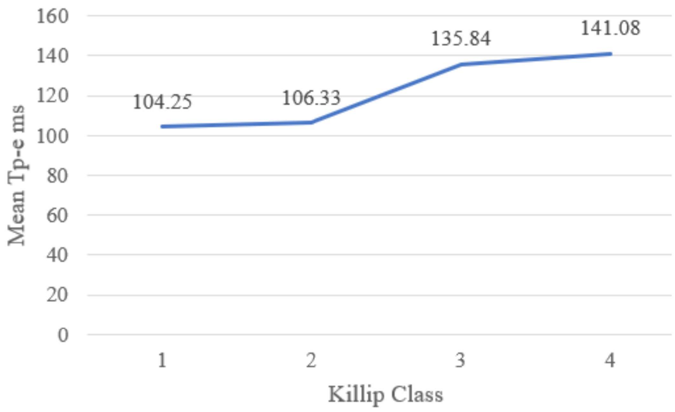

Figure 1A

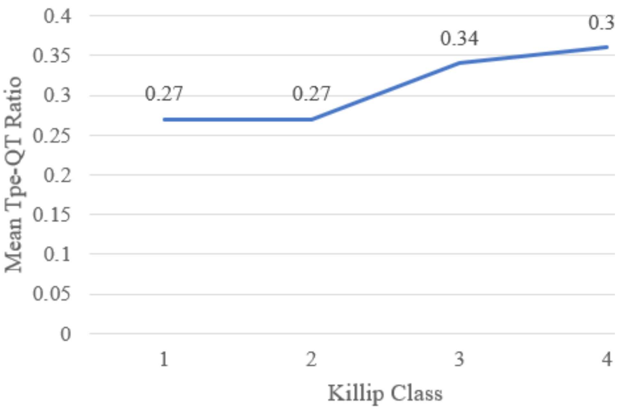

Figure 1B

Supplementary Figure 1: The mean Tp-e interval (1A) and the Tp-e/QT ratio (1B) across the Killip class.

Supplementary Table 1: Descriptive statistics of the Tp-e interval and Tp-e/QT ratio across subgroups

Variable		Tp-e interval (ms)	p value	Tp-e/QT ratio	p value
		Mean±SD		Mean±SD	
Age	<65	108.80±30(116)	0.087	0.28±0.08(116)	0.099
	≥ 65	116.52±32.4(80)		0.30±0.11(80)	
Gender	Male	111.75±30.76(136)	0.891	0.29±0.10(136)	0.628
	Female	112.41±32.02(60)		0.28±0.08(60)	
Diabetes mellitus	Yes	111.13±31.1(87)	0.741	0.28±0.08(87)	0.521
	No	112.60±31.17(109)		0.29±0.10(109)	
Hypertension	Yes	110.95±31.61(119)	0.579	0.28±0.10(119)	0.42
	No	113.49±30.35(77)		0.29±0.08(77)	
Dyslipidemia	Yes	111.22±29.73(115)	0.598	0.28±0.08(115)	0.291
	No	112.98±33.03(81)		0.29±0.11(81)	
Smoking	Yes	113.83±30.82(141)	0.175	0.29±0.08(141)	0.466
	No	107.12±31.46(55)		0.27±0.12(55)	
Alcohol	Yes	109.34±34.93(68)	0.393	0.28±0.12(68)	0.979
	No	113.33±28.58(128)		0.28±0.07(128)	
STEMI	Yes	113.83±31.36(156)	0.094	0.29±0.10(156)	0.091
	No	104.60±29.14(40)		0.26±0.08(40)	
Arrythmia	Yes	121.00±31.42(128)	**<0.001**	0.31±0.10(128)	**<0.001**
	No	94.91±22.07(68)		0.25±0.06(68)	
LVEF	< 40%	133.47±28.15(46)	**<0.001**	0.35±0.08(46)	**<0.001**
	>40%	105.16 28.84(150)		0.27±0.10(150)	
Pulmonary edema	Yes	129.21±32.93(51)	**<0.001**	0.32±0.09(51)	**<0.001**
	No	105.88±28.07(145)		0.27±0.09(145)	
Shock	Yes	133.07±28.03(57)	**<0.001**	0.34±0.09(57)	**<0.001**
	No	103.29±28.02(139)		0.27±0.09(139)	
AKI	Yes	130.78±31.65(63)	**<0.001**	0.33±0.09(63)	**<0.001**
	No	103.03±26.57(133)		0.27±0.09(133)	
Death	Yes	140.83±27.62(16)	**<0.001**	0.38±0.08(16)	**<0.001**
	No	109.38±30.11(180)		0.28±0.09(180)	

AKI, acute kidney injury; LVEF, left ventricular ejection fraction; QTc, corrected QT interval; ms, milliseconds; SD, standard deviation; STEMI, ST-elevation myocardial infarction.

Supplementary Table 2: Distribution of Arrhythmia Subtypes in the Study Population by Survival Status

			Type of Arrhythmia													Total
			1/2 degree heart block	AIVR	Atrial fibrillation	CHB	Junctional rhythm	LBBB	NO ARRHYTHMIA	RBBB	Sinus bradycardia	Sinus tachycardia	Ventricular fibrillation	Ventricular tachycardia	VPC	
Death	No	Count	7	2	9	15	1	3	68	3	26	16	3	19	8	180
		Expecte	6.4	1.8	9.2	14.7	0.9	2.8	62.4	2.8	23.9	17.4	3.7	25.7	8.3	180.

		d Count													0
	Yes	Count	0	0	1	1	0	0	0	0	0	3	1	9	1	16
		Expected Count	0.6	0.2	0.8	1.3	0.1	0.2	5.6	0.2	2.1	1.6	0.3	2.3	0.7	16.0
Total		Count	7	2	10	16	1	3	68	3	26	19	4	28	9	196
		Expected Count	7.0	2.0	10.0	16.0	1.0	3.0	68.0	3.0	26.0	19.0	4.0	28.0	9.0	196.0

AIVR, accelerated idioventricular rhythm; CHB, complete heart block; LBBB, left bundle branch block; RBBB, right bundle branch block; VPC, ventricular premature complexes.

Supplementary Table 3: Distribution of Arrhythmia Subtypes by left ventricular ejection fraction (<40% vs ≥40%)

			Type of Arrhythmia													Total
			1/2 DEGREE	AIVR	Atrial fibrillation	CHB	Junctional rhythm	LBBB	NO ARRHYTHMIA	RBBB	Sinus bradycardia	Sinus tachycardia	Ventricular fibrillation	Ventricular tachycardia	VPC	
LVEF	<40%	Count	0	0	2	9	0	0	5	1	0	5	2	21	2	47
		Expected Count	1.7	0.5	2.4	3.8	0.2	0.7	16.3	0.7	6.2	4.6	1.0	6.7	2.2	47.0
	>40%	Count	7	2	8	7	1	3	63	2	26	14	2	7	7	149
		Expected Count	5.3	1.5	7.6	12.2	0.8	2.3	51.7	2.3	19.8	14.4	3.0	21.3	6.8	149.0
Total		Count	7	2	10	16	1	3	68	3	26	19	4	28	9	196
		Expected Count	7.0	2.0	10.0	16.0	1.0	3.0	68.0	3.0	26.0	19.0	4.0	28.0	9.0	196.0

AIVR, accelerated idioventricular rhythm; CHB, complete heart block; LBBB, left bundle branch block; LVEF, left ventricular ejection fraction; RBBB, right bundle branch block; VPC, ventricular premature complexes.

Supplementary Table 4: Distribution of Arrhythmia Subtype by Time of Onset

	Type of Arrhythmia	Total

			1/2 DEGREE	AIVR	Atrial fibrillation	CHB	Junctional rhythm	LBBB	NO ARRHYTHMIA	RBBB	Sinus bradycardia	Sinus tachycardia	Ventricular fibrillation	Ventricular tachycardia	VPC	
time of onset of arrhythmia	< 6	Count	6	0	5	15	0	3	0	3	21	16	3	19	9	100
		Expected Count	3.6	1.0	5.1	8.2	0.5	1.5	34.7	1.5	13.3	9.7	2.0	14.3	4.6	100.0
	< 12	Count	1	2	2	0	1	0	0	0	0	1	1	5	0	13
		Expected Count	0.5	0.1	0.7	1.1	0.1	0.2	4.5	0.2	1.7	1.3	0.3	1.9	0.6	13.0
	< 48	Count	0	0	3	1	0	0	68	0	5	2	0	4	0	83
		Expected Count	3.0	0.8	4.2	6.8	0.4	1.3	28.8	1.3	11.0	8.0	1.7	11.9	3.8	83.0
Total		Count	7	2	10	16	1	3	68	3	26	19	4	28	9	196
		Expected Count	7.0	2.0	10.0	16.0	1.0	3.0	68.0	3.0	26.0	19.0	4.0	28.0	9.0	196.0

AIVR, accelerated idioventricular rhythm; CHB, complete heart block; LBBB, left bundle branch block; RBBB, right bundle branch block; VPC, ventricular premature complexes.

Supplementary Table 5A: Age Groups and Arrhythmia Occurrence

**Age group (years)**	**Arrhythmia present n%**	**Arrhythmia absent n%**	**Total n %**
**40-50**	14 (58.3)	10 (41.7)	24 (12.2)
**51-60**	27 (40.3)	40 (59.7)	67 (34.2)
**61-70**	15 (24.6)	46 (75.4)	61 (31.1)
**71-80**	10 (27.8)	26 (72.2)	36 (18.4)
**81-90**	2 (25.0)	6 (75.0)	8 (4.1)
**Total**	**68 (34.7)**	**128 (65.3)**	**196 (100)**

Supplementary Table 5B: Correlation of age with arrythmia occurrence

	Value	df	Asymptotic Significance (2-sided)
Pearson Chi-Square	10.689	4	0.030

Likelihood Ratio	10.516	4	0.033
Linear-by-Linear Association	7.635	1	0.006
N of Valid Cases	196		

df -degree of freedom

Supplementary Table 6: Receiver operating characteristic (ROC) curve analysis of Tpeak–Tend (Tp–e) interval for predicting arrhythmia occurrence.

**Parameter**	**Value**
Test variable	Tp–e interval (ms)
Area under the curve (AUC)	0.736
Standard error	0.035
95% Confidence interval	0.666 – 0.805
p value	< 0.001

Supplementary Table 7: Association of beta-blocker with arrythmia

Beta blocker use	Arrhythmia absent	Arrhythmia present
No	19 (29.7%)	64 (52.5%)
Yes	45 (70.3%)	58 (47.5%)
Total	64 (100%)	122 (100%)
Chi-Square p-value = 0.003		

Supplementary Table 8: Association of beta-blocker with mortality

Beta blocker use	Survival	Mortality
No	68 (39.8%)	15(100%)
Yes	103 (60.2%)	0 (0%)
Total	171 (100%)	15 (100%)
Fischer's exact test p-value <0.001		

Supplementary Table 9: Association of reperfusion therapy with arrythmia

Reperfusion therapy	Arrhythmia absent	Arrhythmia present
No	13 (20.3%)	28 (23%)
Yes	51 (79.7%)	94(77%)
Total	64 (100%)	122 (100%)
Chi-Square p-value = 0.680		

Supplementary Table 10: Association of reperfusion therapy with mortality

Reperfusion therapy	Survival	Mortality
No	36 (21.1%)	5 (33.3%)
Yes	135 (78.9%)	10 (66.7%)
Total	171(100%)	15 (100%)
Chi-Square p-value = 0.271		
